# Supplementary material for: A Cas3-base editing tool for targetable in vivo mutagenesis
Source: Nat Commun. 2023 Jun 9;14:3389. doi: 10.1038/s41467-023-39087-z (PMC10256805; doi:10.1038/s41467-023-39087-z)
Supplement: Supplementary file 1 — Supplementary Information File [file 41467_2023_39087_MOESM1_ESM.pdf]

Supplementary Information File

A Cas3-base editing tool for targetable *in vivo* mutagenesis

Anna Zimmermann[1,2], Julian E. Prieto-Vivas[1,2], Charlotte Cautereels[1,2], Anton Gorkovskiy[1,2], Jan Steensels[1,2], Yves Van de Peer[3,4,5,6*] and Kevin J. Verstrepen[1,2,*]

[1]VIB Laboratory for Systems Biology, VIB-KU Leuven Center for Microbiology, Leuven, 3001, Belgium

[2]Laboratory for Genetics and Genomics, Center of Microbial and Plant Genetics, Department M2S, KU Leuven, Gaston Geenslaan 1, 3001, Leuven, Belgium

[3]Department of Plant Biotechnology and Bioinformatics, Ghent University, Ghent, Belgium

[4]VIB Center for Plant Systems Biology, Ghent, Belgium

[5]Department of Biochemistry, Genetics and Microbiology, University of Pretoria, Pretoria, South Africa

[6]College of Horticulture, Academy for Advanced Interdisciplinary Studies, Nanjing Agricultural University, 210095, Nanjing, China

[*]Correspondence: yves.vandepeer@psb.ugent.be kevin.verstrepen@kuleuven.be

This PDF file includes:

- Supplementary Figures 1-12
- Supplementary Tables 1-8

Supplementary Figures

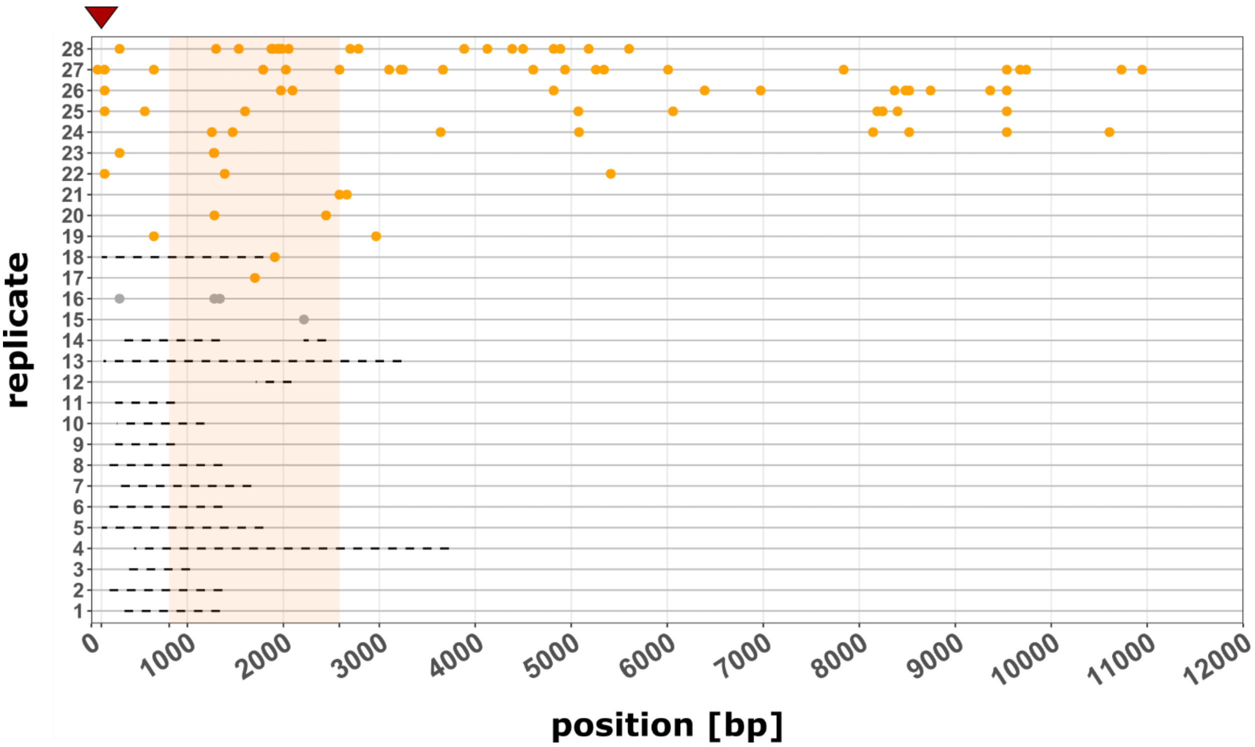

Supplementary Fig. 1 Mutations introduced by Cas3-APOBEC1 targeted to target site 2.
Position of mutations introduced within a 12 kb window in each biological replicate of the strain expressing Cas3-APOBEC1 targeted to target site 2. Cytidine deaminations are represented by orange circles, deletions by a dashed line and other SNVs by grey circles. For easier comparison with Cas3-APOBEC1 targeted to target site 1 and untargeted Cas3-APOBEC1, data are shown in the same format as in Fig. 3c. The red triangle indicates target site 2 and the shaded orange box indicates the position of the *CAN1* marker gene. Source data for this figure are provided as a Source Data file.

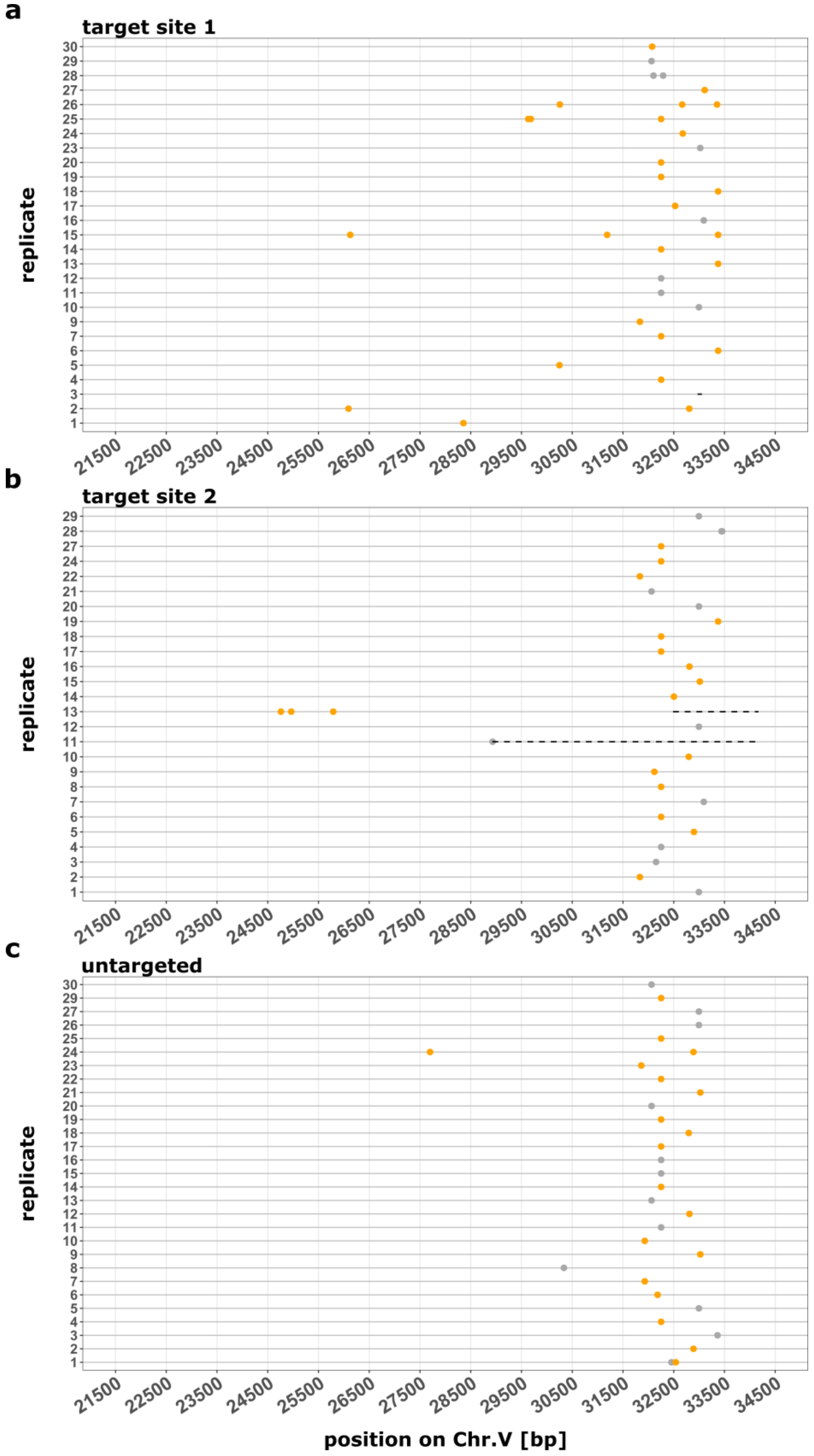

Supplementary Fig. 2 Mutations introduced by Cas3-CDA1 within 12 kb window.
Position of mutations introduced within a 12 kb window in each biological replicate of the strain expressing Cas3-CDA1 targeted to **a** target site 1, **b** target site 2 and **c** untargeted. Cytidine deaminations are represented by orange circles, deletions by a dashed line and other SNVs by grey circles. Source data for this figure are provided as a Source Data file.

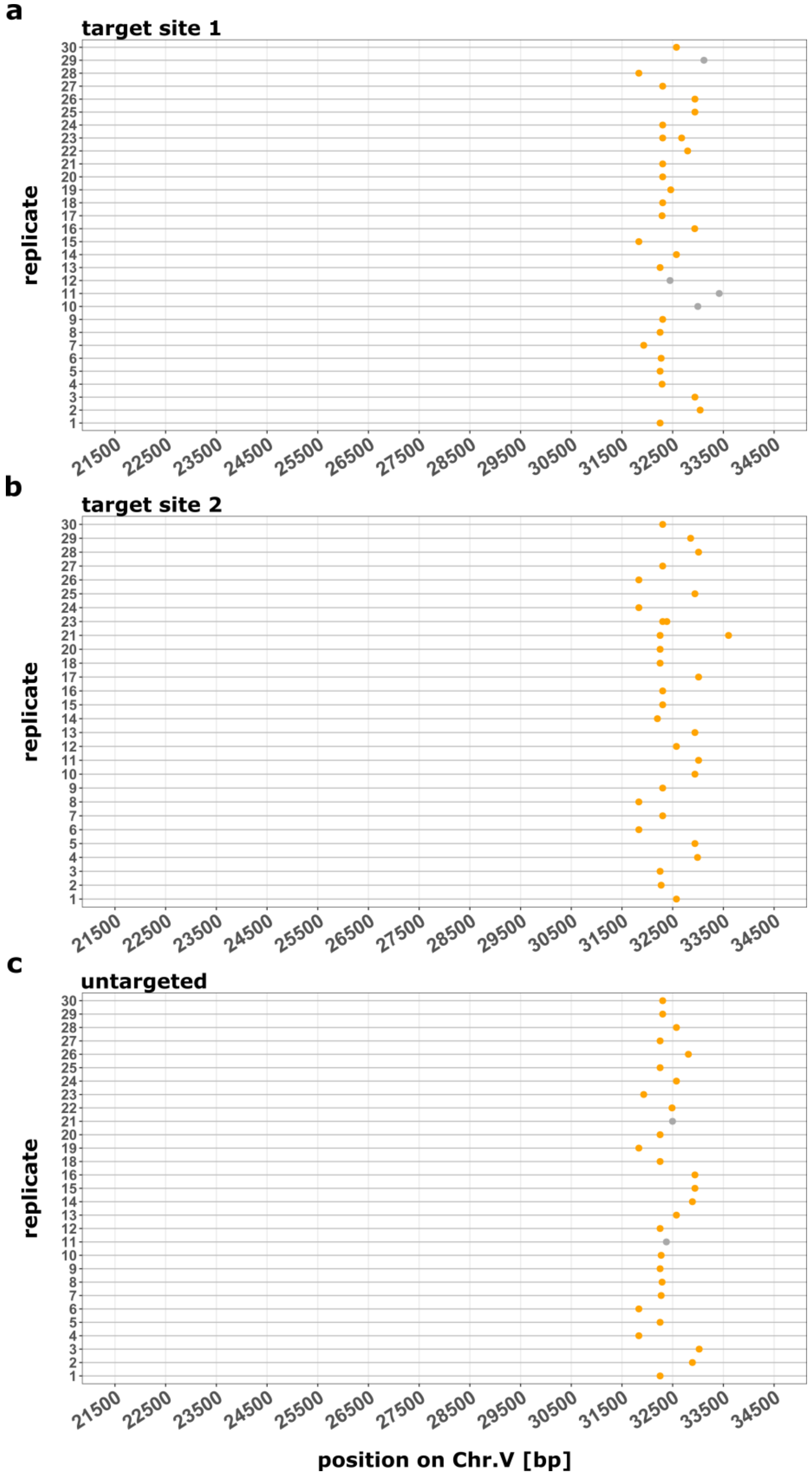

Supplementary Fig. 3 Mutations introduced by dnCas3-APOBEC1 within 12 kb window.
Position of mutations introduced within a 12 kb window in each biological replicate of the strain expressing dnCas3-APOBEC1 targeted to **a** target site 1, **b** target site 2 and **c** untargeted. Cytidine deaminations are represented by orange circles, deletions by a dashed line and other SNVs by grey circles. Source data for this figure are provided as a Source Data file.

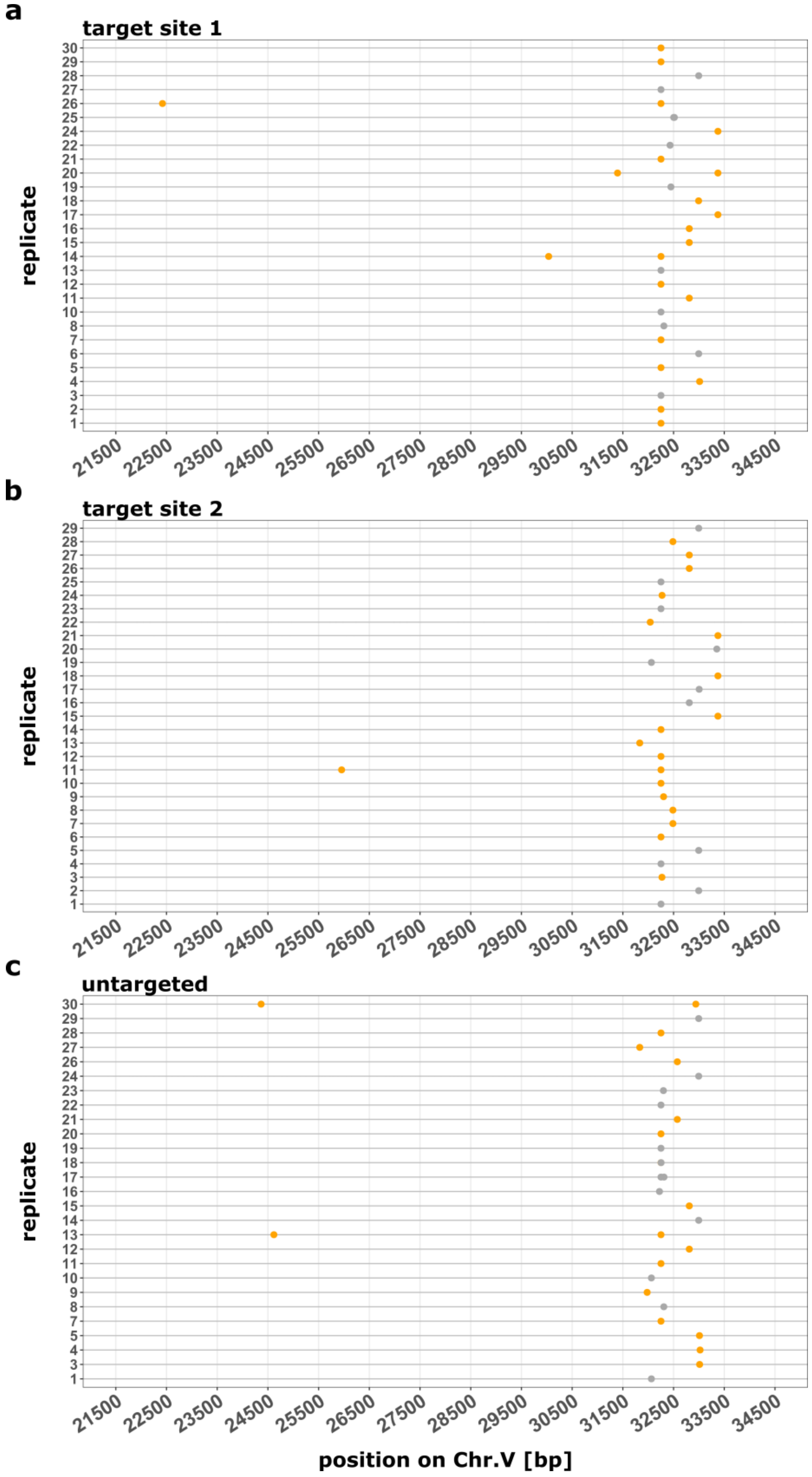

Supplementary Fig. 4 Mutations introduced by dnCas3-CDA1 within 12 kb window.

Position of mutations introduced within a 12 kb window in each biological replicate of the strain expressing dnCas3-CDA1 targeted to **a** target site 1, **b** target site 2 and **c** untargeted. Cytidine deaminations are represented by orange circles, deletions by a dashed line and other SNVs by grey circles. Source data for this figure are provided as a Source Data file.

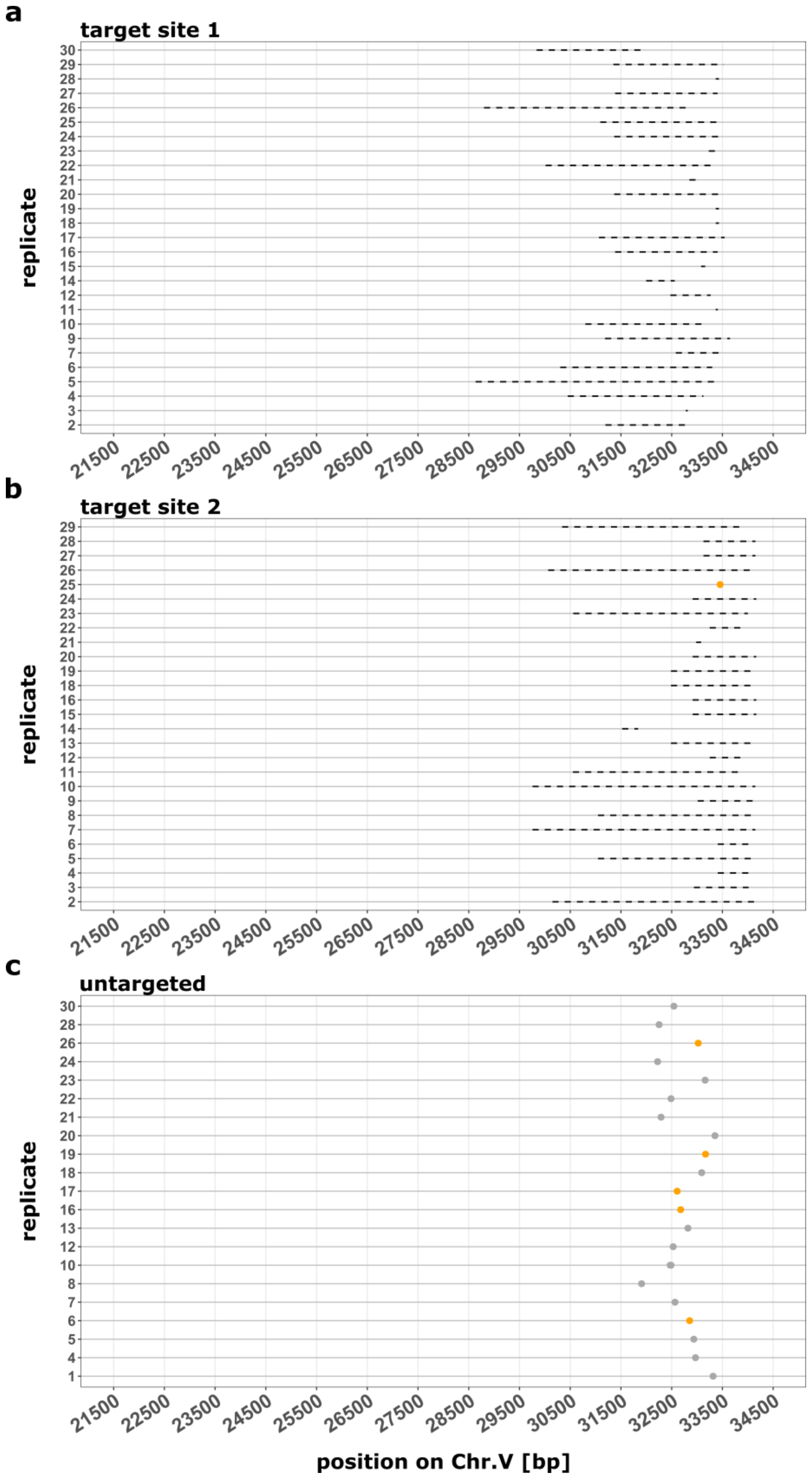

Supplementary Fig. 5 Mutations introduced by unfused Cas3 within 12 kb window.

Position of mutations introduced within a 12 kb window in each biological replicate of the strain expressing Cas3 targeted to **a** target site 1, **b** target site 2 and **c** untargeted. Cytidine deaminations are represented by orange circles, deletions by a dashed line and other SNVs by grey circles. Source data for this figure are provided as a Source Data file.

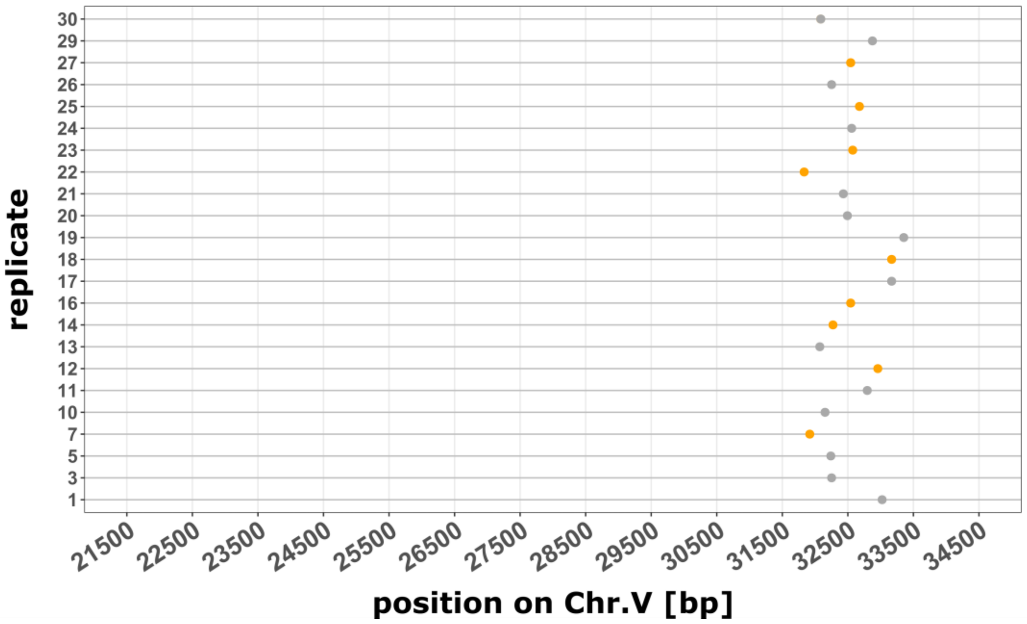

Supplementary Fig. 6 **Mutations introduced by S288c-CB within 12 kb window.**
Position of mutations introduced within a 12 kb window in each biological replicate of the S288c-CB control strain. Cytidine deaminations are represented by orange circles, deletions by a dashed line and other SNVs by grey circles. Source data for this figure are provided as a Source Data file.

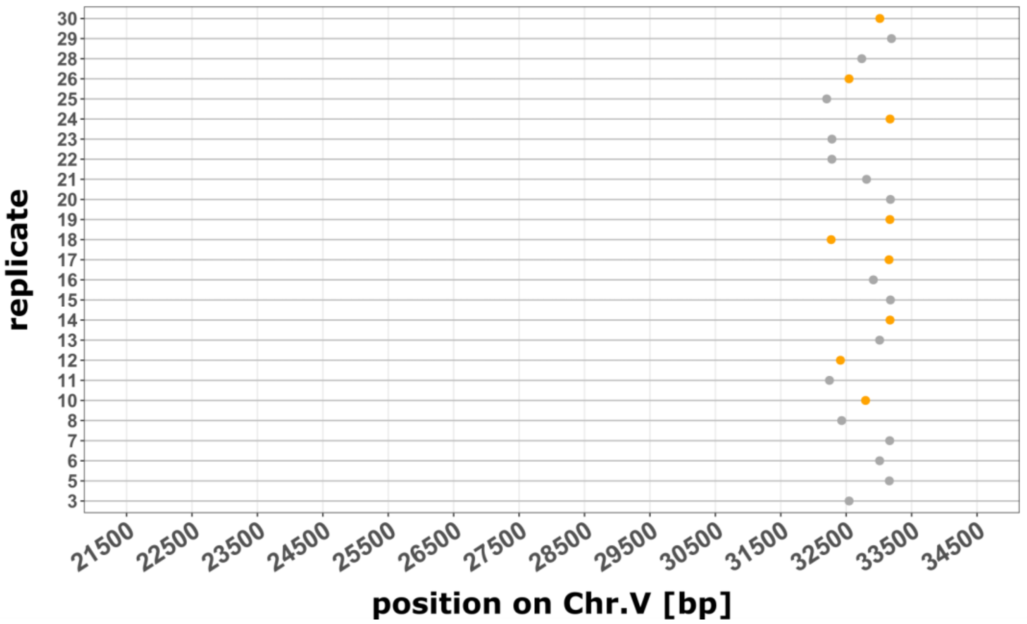

Supplementary Fig. 7 **Mutations introduced by S288c within 12 kb window.**
Position of mutations introduced within a 12 kb window in each biological replicate of the S288c control strain. Cytidine deaminations are represented by orange circles, deletions by a dashed line and other SNVs by grey circles. Source data for this figure are provided as a Source Data file.

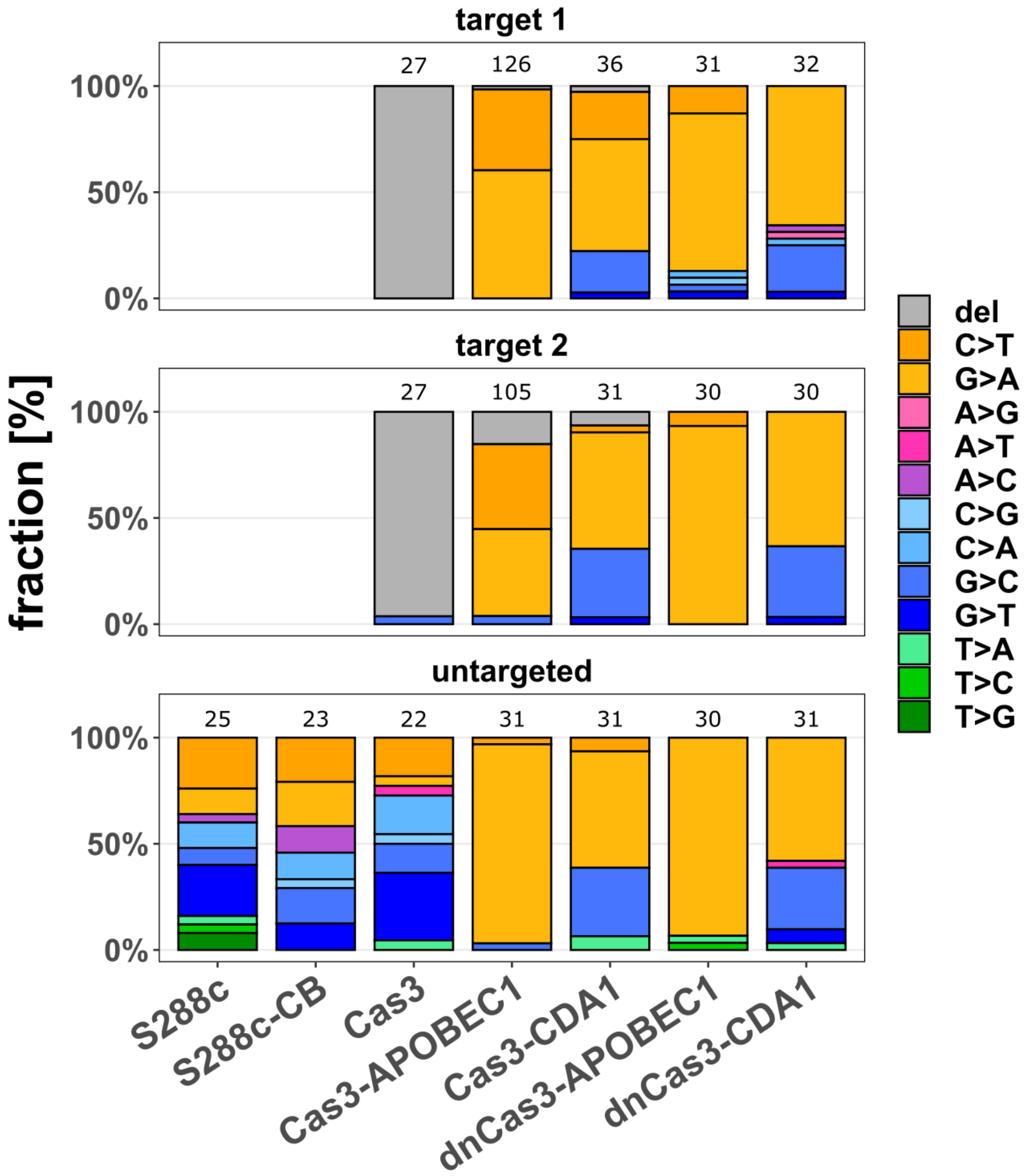

Supplementary Fig. 8 **Proportions of introduced mutations confirm effectiveness of the Cas3-APOBEC1 base-editor.** The fractions for all types of mutations detected within a 12 kb window downstream of the respective target site for strains expressing the four Cas3-base editors, unfused Cas3 and two control strains (S288c and S288c-CB) targeted to target site 1 or 2 or untargeted. The number above each bar shows the total number of mutations detected in all analyzed replicates (n = 21-30) per strain and target site (see Methods and Supplementary Table 8 for information on replicate number). Source data for this figure are provided as a Source Data file.

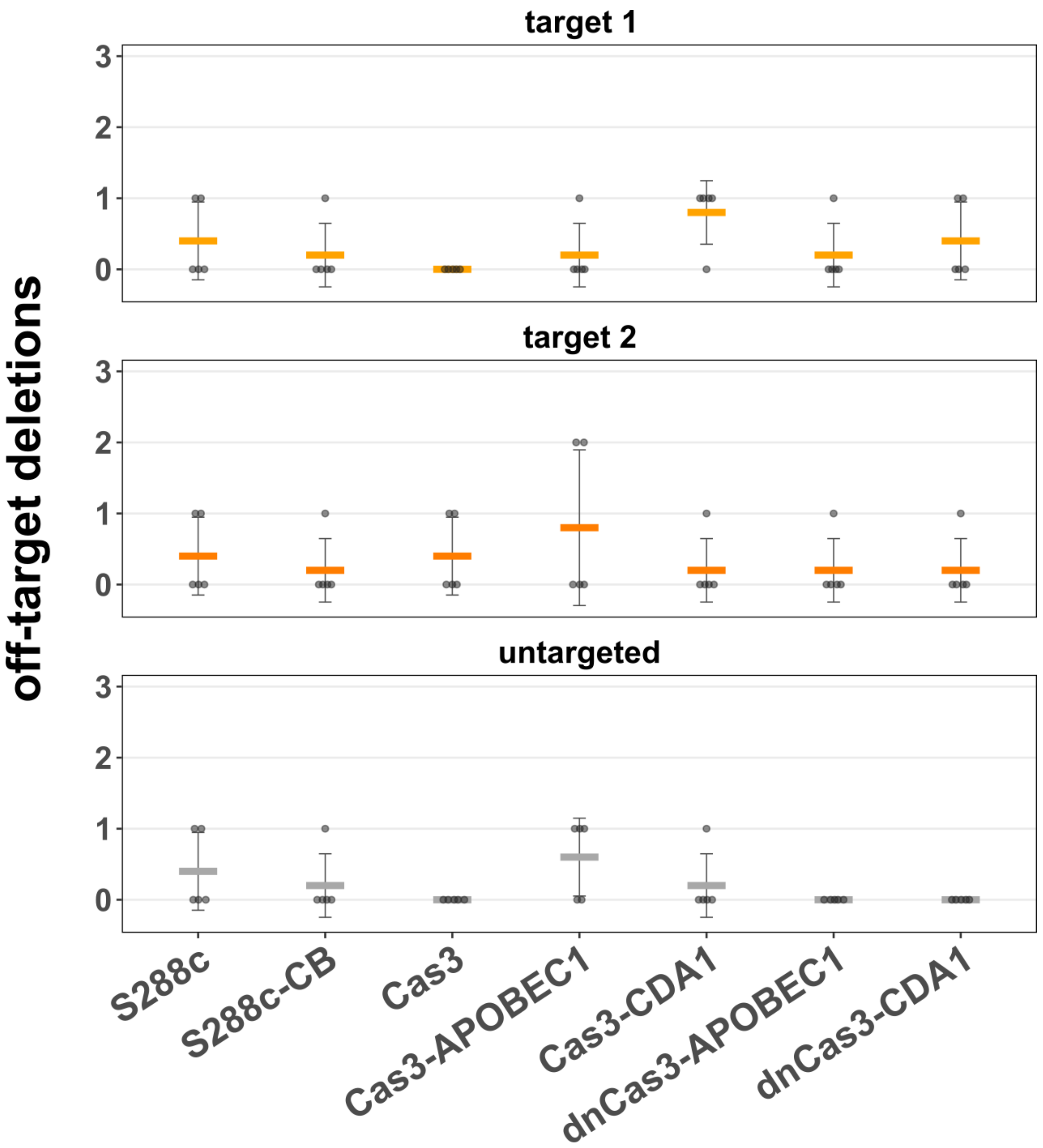

Supplementary Fig. 9 Cas3 base-editors do not cause off-target deletions.

The graph shows the average number of off-target deletions, determined via whole genome sequencing, of strains expressing the four Cas3-base editors, unfused Cas3 and two control strains (S288c and S288c-CB) targeted to target site 1 or 2 or untargeted. Data are presented as mean (horizontal lines) ± SD of n = 5 independent biological replicates (points) per strain and target site. Source data for this figure are provided as a Source Data file.

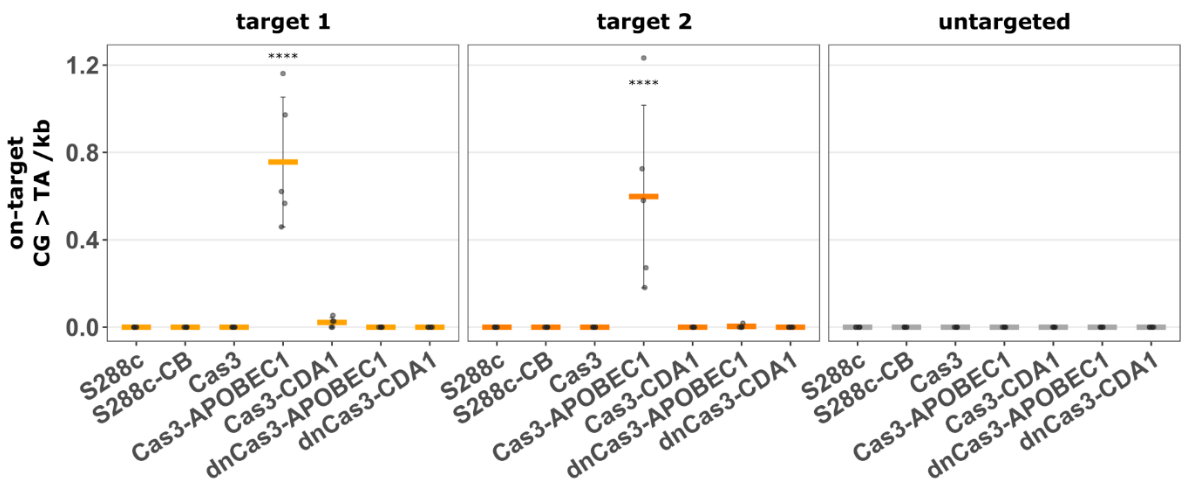

Supplementary Fig. 10 Average number of on-target cytidine deaminations per kb for each strain and target site. Number of cytidine deaminations in the target region (on-target) were determined by whole genome sequencing of strains expressing one of the four different Cas3-base editors, unfused Cas3 and two control strains (S288c and S288c-CB) targeted to either target site 1 or 2, or untargeted. For each strain and target site, five colonies selected from canavanine-containing plates were analyzed. The target region is defined as region on chromosome 5 in which 99.3% of cytidine deaminations were observed in strains expressing Cas3-APOBEC1. Strains S288c and S288c-CB are always untargeted, as they do not contain a crRNA cassette. For easier comparison their respective averages are included in the graphs for target site 1 and 2. Data are presented as mean (horizontal lines) ± SD of n = 5 independent biological replicates (points) per strain and target site. Data were fitted using a generalized linear mixed-effects model. A two-sided post-hoc test with Sidak adjustment was used to identify strains with significant difference to the control strain (S288c), ****$p \leq 0.0001$. Specifically, p-values are 5.18×10^{-11} for Cas3-APOBEC1 target site 1, 7.58×10^{-6} for Cas3-APOBEC1 target site 2, and 1 for all other samples. Source data for this figure are provided as a Source Data file.

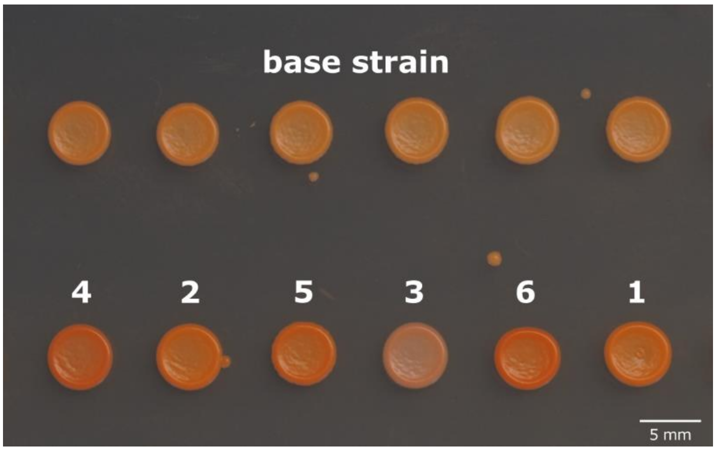

Supplementary Fig. 11 Mutated strains show more intense coloration compared to the base strain. Strains selected for more intense red coloration (strain 1-6, bottom row) are compared to the non-mutagenized base strain (upper row). The base strain was plated above each mutated strain for easier comparison. Each strain was grown for 36 hours in SC (+2% glucose). Cultures were adjusted to same density and subsequently plated on SC (+2% glucose) medium. The picture was taken after 72 hours of growth at 30 °C. Scale bar represents 5 mm.

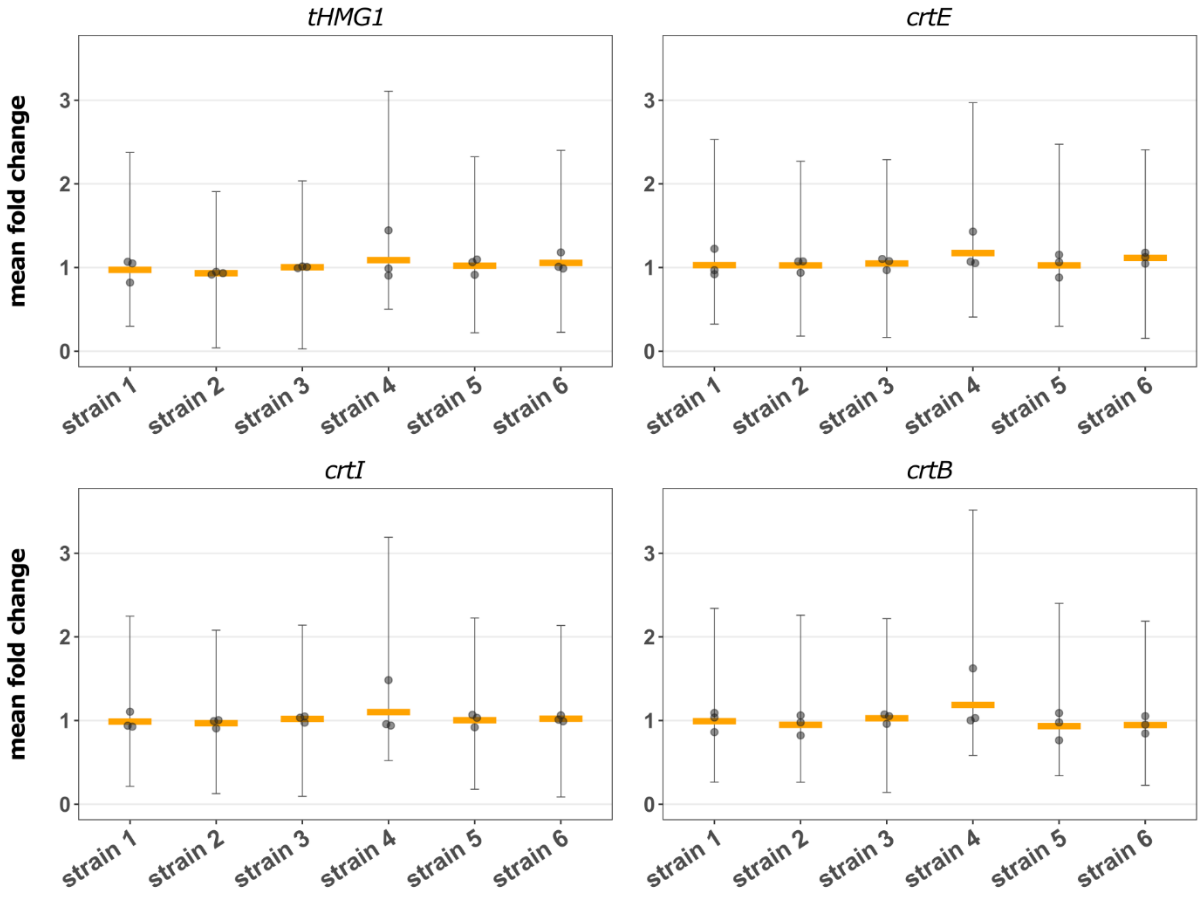

Supplementary Fig. 12 Mutated strains and base strain do not show significant differences in lycopene gene expression. To assess whether mutations introduced in the lycopene pathway of the selected strains effect gene expression, the relative expression of the introduced genes (*tHMG1*, *crtE*, *crtI*, *crtB*) was compared to the respective gene expression in the base strain. Relative gene expression was measured via qPCR using *TAF10* and *ALG9* as reference genes (see methods). a-d Mean fold change of *tHMG1*, *crtE*, *crtI* and *crtB* expression in selected strains 1-6 relative to the base strain. Data are presented as mean (orange lines) ± 95% CI of N = 3 independent biological replicates (points). Data were fitted using a linear model and a two-sided post-hoc test with Dunnettx adjustment was used to identify strains with significant difference to the base strain ($p \leq 0.05$). Exact p-values can be found in Supplementary Data 1, p-values. Source data for this figure are provided as a Source Data file.

Supplementary Tables

Supplementary Table 1. Average number of colonies on selective medium

Average number of colonies on canavanine-containing medium after inducing or non-inducing growth conditions for strains expressing the four Cas3-base editors, unfused Cas3 and two control strains (S288c and S288c-CB) targeted to either target site 1 or 2, or untargeted. Averages are given for n = 5 independent biological replicates per strain and target site.

Strain	Target	Induced		Uninduced	
		Average	SD	Average	SD
S288c	untargeted	7.0	7.6	1.6	1.5
S288c-CB	untargeted	5.8	4.3	3.8	3.6
Cas3	target 1	1253.2	276.5	8.2	6.5
Cas3	target 2	1583.4	524.3	17.8	13.3
Cas3	untargeted	15.4	11.7	13.0	5.6
Cas3-APOBEC1	target 1	3365.2	652.2	13.6	18.7
Cas3-APOBEC1	target 2	1422.8	1015.7	25.4	15.5
Cas3-APOBEC1	untargeted	370.4	23.4	29.0	16.9
Cas3-CDA1	target 1	2434.8	515.3	226.8	122.2
Cas3-CDA1	target 2	1447.6	347.5	314.0	245.0
Cas3-CDA1	untargeted	776.8	258.9	175.4	55.3
dnCas3-APOBEC1	target 1	203.4	32.8	13.6	23.8
dnCas3-APOBEC1	target 2	196.4	20.7	12.6	3.4
dnCas3-APOBEC1	untargeted	154.2	40.2	7.0	6.2
dnCas3-CDA1	target 1	379.0	71.0	60.0	24.4
dnCas3-CDA1	target 2	595.8	163.4	89.8	42.5
dnCas3-CDA1	untargeted	314.4	39.9	72.2	22.2

Supplementary Table 2. Number of NPPM 481-resistant colonies

The number of resistant colonies was analyzed for strains expressing the Cas3-APOBEC1 base editor targeted 386 bp downstream of the *SEC14* stop codon, a control strain expressing untargeted Cas3-APOBEC1 and the parental strain CEN.PK-CB. Each strain was grown in 3 independent biological replicates (rep. 1, rep. 2, rep. 3). Each biological replicate was plated in 3 technical replicates (plate 1, plate 2 plate 3) on SC-Glu medium supplemented with 3 µM NPPM 481 (~50.000 cells/plate plated). Colony formation was assessed after 48 hours of incubation at 30 °C.

	CEN.PK-CB			Cas3-APOBEC1 (targeted)			Cas3-APOBEC1 (untargeted)		
	Rep. 1	Rep. 2	Rep. 3	Rep. 1	Rep. 2	Rep. 3	Rep. 1	Rep. 2	Rep. 3
**Plate 1**	0	0	0	10	8	13	1	2	2
**Plate 2**	0	1	0	7	10	8	3	1	2
**Plate 3**	1	0	0	11	12	11	2	1	1

Supplementary Table 3. Mutations identified in *SEC14* locus

Mutations were identified via Sanger sequencing of a 1839 bp amplicon ranging from 171 bp upstream to 597 bp downstream of the *SEC14* start codon. Amplicons were amplified from colonies able to grow on 3 µM NPPM 481-containing medium after induction of the CoMuTER system (Cas3-APOBEC1, targeted). CDS, coding sequence.

**Colony**	**Mutation**	**Position rel. to ATG [bp]**	**Type**
1	GTC > ATC	+616	V154I
2	C > T	-68	upstream CDS
2	C > T	+51	Intron
2	C > T	+88	Intron
2	C > T	+112	Intron
2	TTC > TTT	+474	F106F
2	TCA > TTA	+674	S173L
3	GAA > AAA	+604	E150K
4	C > T	+1249	downstream CDS
4	C > T	+1252	downstream CDS
5	GGT > AGT	+784	G210S
6	GAA > AAA	+604	E150K
7	TCA > TTA	+674	S173L
7	TCT > TTT	+821	S222F
7	C > T	+1249	downstream CDS
7	C > T	+1252	downstream CDS
8	TCA > TTA	+674	S173L
9	GTC > ATC	+616	V154I
10	TTC > TTT	+474	F106F
10	TCC > TTC	+704	S183F
11	C > T	+112	Intron
11	TCA > TTA	+674	S173L
11	TTC > TTT	+819	F221F
11	TCT > TTT	+821	S222F
11	C > T	+1249	downstream CDS
11	C > T	+1252	downstream CDS
11	C > T	+1253	downstream CDS
12	GAA > AAA	+604	E150K
13	TTG > TTA	+342	L62L
13	GAA > AAA	+604	E150K
14	GTC > ATC	+616	V154I
15	GAA > AAA	+604	E150K
16	CAT > TAT	+490	H112Y
17	C > T	+112	Intron
17	C > T	+1252	downstream CDS
18	TCA > TTA	+674	S173L
18	C > T	+1252	downstream CDS

Supplementary Table 4. List of strains used in this study

Name in this study	Number	Genotype	Reference
S288c	KV172	prototrophic haploid S288c, *MATα*	FY5 from[1]
S288c-CB	AZ_142	KV172 containing Cascade subunits; Cas5, Cas6, Cas7, Cas8, Cas11	This study
Cas3-CDA1	AZ_144	AZ_142 harboring plasmid pCas3-CDA1	This study
dnCas3-CDA1	AZ_146	AZ_142 harboring plasmid pdnCas3-CDA1	This study
Cas3-APOBEC1	AZ_147	AZ_142 harboring plasmid pCas3-APOBEC1	This study
dnCas3-APOBEC1	AZ_149	AZ_142 harboring plasmid pdnCas3-APOBEC1	This study
Cas3	AZ_150	AZ_142 harboring plasmid pCas3	This study
CEN.PK	KV3012	CEN.PK2-1C / *MATa ura3 leu2 trp1 his3*	From[2]
CEN.PK-CB	AZ_258	KV3012 containing Cascade subunit genes; *Cas5*, *Cas6*, *Cas7*, *Cas8*, *Cas11*	This study
Lycopene base strain	AZ_293	AZ_258 containing lycopene pathway genes (*tHMG1*, *crtI*, *crtE*, *crtB*)	This study
Lycopene base strain + Cas3-base editor	AZ_295	AZ_293 harboring pCas3-APOBEC1	This study
Lycopene strain 1	AZ_301	AZ_258 containing mutated lycopene pathway*	This study
Lycopene strain 2	AZ_297	AZ_258 containing mutated lycopene pathway*	This study
Lycopene strain 3	AZ_299	AZ_258 containing mutated lycopene pathway*	This study
Lycopene strain 4	AZ_296	AZ_258 containing mutated lycopene pathway*	This study
Lycopene strain 5	AZ_298	AZ_258 containing mutated lycopene pathway*	This study
Lycopene strain 6	AZ_300	AZ_258 containing mutated lycopene pathway*	This study

*Mutations can be found in the Source Data file, Figure 5d.

Supplementary Table 5. Integration sites of Cascade subunits

Cascade subunit	Integration site in S288c genome (assembly R64)
Cse1	ChrVI: 233320
Cse2	ChrVII: 508455
Cas5	ChrII: 237457
Cas6	ChrIII: 113821
Cas7	ChrIV: 463147

Supplementary Table 6. List of CRISPR-Cas9 guide RNAs used in this study

gRNA	Info	Sequence 5'->3'
gRNA_Cas8_fv	gRNA to integrate Cascade subunit Cas8	ATGAAAGACAACTATAGGGC
gRNA_Cas8_rv		GCCCTATAGTTGTCTTTCAT
gRNA_Cas11_fv	gRNA to integrate Cascade subunit Cas11	CAACAATTGTTACAATAGTA
gRNA_Cas11_rv		TACTATTGTAACAATTGTTG
gRNA_Cas5_fv	gRNA to integrate Cascade subunit Cas5	GTCCGCTAAACAAAAGATCT
gRNA_Cas5_rv		AGATCTTTTGTTTAGCGGAC
gRNA_Cas6_fv	gRNA to integrate Cascade subunit Cas6	CACTTGTCAAACAGAATATA
gRNA_Cas6_rv		TATATTCTGTTTGACAAGTG
gRNA_Cas7_fv	gRNA to integrate Cascade subunit Cas7	TAGTGCACTTACCCCACGTT
gRNA_Cas7_rv		AACGTGGGGTAAGTGCACTA
gRNA_lyc_fw	gRNA to integrate lycopene biosynthesis cassette 436 bp upstream of *CAN1* in CEN.PK-CB	AGAAATCTAGGGTTTCTGTG
gRNA_lyc_rv		CACAGAAACCCTAGATTTCT

Supplementary Table 7. List of Cas3 crRNA spacer sequences used in this study.
All crRNAs target sites are located directly downstream of a 5'-AAG-3' PAM.

**crRNA**	**Info**	**Sequence 5'->3'**
crRNA_target_site_1_fw	crRNA targets 68 bp downstream of *CAN1* start codon	AGGGTTGTGACCGGCTCATTGTACATATGCTT
crRNA_target_site_1_rv		AAGCATATGTACAATGAGCCGGTCACAACCCT
crRNA_target_site_2_fw	crRNA targets 675 bp upstream of *CAN1* start codon	AGTTCTTACTCAGTGTGAACGTGTTCTAAATA
crRNA_target_site_2_rv		TATTTAGAACACGTTCACACTGAGTAAGAACT
crRNA_lyc_fw	crRNA targets directly upstream of lycopene biosynthesis cassette	CAACGGTAGCAGCTGGTACGATTTTTACTCAT
crRNA_lyc_rv		ATGAGTAAAAATCGTACCAGCTGCTACCGTTG
crRNA_sec14_fw	crRNA targets 386 bp downstream of SEC14 stop codon	AGTATTCATTAGAAGTACAATGGTAGCCCTAT
crRNA_sec14_rv		ATAGGGCTACCATTGTACTTCTAATGAATACT

Supplementary Table 8. Number of analyzed replicates per strain and target site after Nanopore sequencing. The table shows the number of replicates used in sequencing analysis of a 12 kb window downstream of the respective target site of strains expressing the four Cas3-base editors, unfused Cas3 and the two control strains (S288c and S288c-CB) targeted to target site 1 or 2 or untargeted.

**Strain**	**Target site**	**Number of biological replicates**
Cas3-APOBEC1	target site 1	29
	target site 2	28
	untargeted	30
Cas3-CDA1	target site 1	27
	target site 2	26
	untargeted	29
dnCas3-APOBEC1	target site 1	30
	target site 2	28
	untargeted	29
dnCas3-CDA1	target site 1	28
	target site 2	29
	untargeted	27
Cas3	target site 1	27
	target site 2	27
	untargeted	21
S288c-CB	untargeted	23
S288c	untargeted	25

References

1. Brachmann, C. B. *et al.* Designer deletion strains derived from Saccharomyces cerevisiae S288C. A useful set of strains and plasmids for PCR-mediated gene disruption and other applications. *Yeast (Chichester, England)* **14,** 115–132; 10.1002/(SICI)1097-0061(19980130)14:2<115::AID-YEA204>3.0.CO;2-2 (1998).

2. Entian, K.-D. & Kötter, P. 25 Yeast Genetic Strain and Plasmid Collections. In *Methods in Microbiology : Yeast Gene Analysis,* edited by I. Stansfield & M. Stark, JR (Academic Press2007), Vol. 36, pp. 629–666.
