# Supplementary material for: A Cas3-base editing tool for targetable in vivo mutagenesis
Source: Nat Commun. 2023 Jun 9;14:3389. doi: 10.1038/s41467-023-39087-z (PMC10256805; doi:10.1038/s41467-023-39087-z)
Supplement: Supplementary file 3 — Description of Additional Supplementary Files [file 41467_2023_39087_MOESM3_ESM.pdf]

Title: Supplementary Data 1

Description:

Sheet 1 – Primers - List of primers used in this study
Sheet 2 – Cas3-fusion constructs - Sequences and design of Cas3-base editors and unfused Cas3
Sheet 3 – crRNA cassette - Sequence and design of crRNA cassette
Sheet 4 – Cas3-base editor plasmids - Full sequences of Cas3-base editor plasmids
Sheet 5 – Cascade subunits - Sequence and design of Cascade subunits
Sheet 6 – WGS SNVs – all SNVs obtained after WGS data analysis
Sheet 7 – WGS INDELs - all INDELs obtained after WGS data analysis
Sheet 8 – Primers lycopene cassette - Primers used to generate overlapping gene expression cassettes of
individual lycopene biosynthesis gene constructs
Sheet 9 – Lycopene cassette - Sequence and design of introduced lycopene cassette
Sheet 10 – Raw CT values - Raw CT values of qPCR
Sheet 11 – p-values - All p-values obtained through statistical analysis of data presented in this study
